# Effect of Conditioner Type and Downforce, and Pad Surface Micro-Texture on SiO_2_ Chemical Mechanical Planarization Performance

**DOI:** 10.3390/mi10040258

**Published:** 2019-04-18

**Authors:** Jeffrey McAllister, Calliandra Stuffle, Yasa Sampurno, Dale Hetherington, Jon Sierra Suarez, Leonard Borucki, Ara Philipossian

**Affiliations:** 1 Department of Chemical and Environmental Engineering, University of Arizona, Tucson, AZ 85721, USA; calliandra@email.arizona.edu (C.S.); yasayap@email.arizona.edu (Y.S.); ara@email.arizona.edu (A.P.); 2 Araca Incorporated, Tucson, AZ 85718, USA; 3 Sandia National Laboratories, Albuquerque, NM 87116, USA; dalehetherington@email.arizona.edu (D.H.); jasierr@sandia.gov (J.S.S.); lborucki@aracainc.com (L.B.)

**Keywords:** chemical mechanical planarization, SiO_2_, pad surface micro-texture, conditioner disc

## Abstract

Based on a previous work where we investigated the effect of conditioner type and downforce on the evolution of pad surface micro-texture during break-in, we have chosen certain break-in conditions to carry out subsequent blanket SiO_2_ wafer polishing studies. Two different conditioner discs were used in conjunction with up to two different conditioning downforces. For each disc-downforce combination, mini-marathons were run using SiO_2_ wafers. Prior to polishing, each pad was broken-in for 30 min with one of the conditioner-downforce combinations. The goal of this study was to polish wafers after this break-in to see how the polishing process behaved immediately after break-in. One of the discs used in this study produced similar micro-texture results at both downforces, which echoed the results seen in the mini-marathon. When comparing the different polishing results obtained from breaking-in the pad with the different discs used in this study, the coefficient of friction (COF) and SiO_2_ removal rate (RR) were uncorrelated in all cases. However, the use of different discs resulted in different COF and RR trends. The uncorrelated COF and RR, as well as the differing trends, were explained by pad micro-texture results (i.e. the differing amount of fractured, poorly supported pad asperity summits).

## 1. Introduction

Chemical mechanical planarization (CMP) is the only economically-viable means of achieving global and local planarization across the wafer surface in integrated circuit manufacturing. CMP involves a rotating polishing pad, typically made of porous polyurethane, being placed into intimate contact with a rotating wafer. An abrasive containing slurry is dispensed onto the surface of the pad, where the pores and groves on the pad surface, combined with the kinematics of the system, transport the slurry to the pad/wafer interface [1]. During CMP, pad micro-texture progressively becomes degraded which can negatively impact polish performance through removal rate (RR) drift, higher within-wafer non-uniformity and higher wafer-level defects. Such degradation in performance impacts CMP module productivity and yield thereby straining work-in-progress and factory output. To counteract this degradation, a rotating diamond disc conditioner is brought into contact with the pad in a similar fashion as the wafer. The conditioner also acts to break-in new pads prior to polishing as it takes some time for the surface micro-texture of a brand-new pad to reach steady-state (the point at which the pad surface micro-texture no longer changes significantly with conditioning time) and meet the stringent demands of modern-day CMP. While being essential, pad break-in decreases polisher availability and wafer throughput. Additionally, it has an impact on the number of dummy wafers that must be run. As such, it is important to improve our understanding of how the polishing process behaves immediately after break-in. Extensive research has shown that pad surface micro-texture plays an important role in CMP and is affected by several factors such as conditioner type, conditioning downforce, and pad break-in time [2,3,4,5,6,7,8,9,10]. In addition, laser scanning confocal microscopy has been successfully used to analyze pad surface micro-texture and has led to a better understanding of how it affects CMP performance [2,3,4,5,6,7,8,9,10]. Sun et al. studied the effect of conditioner type and conditioning downforce on pad topography and found that the surface becomes more abrupt and copper removal rates decrease with increasing conditioning downforce [2]. However, in that case, white light interferometry (which was considered to be a less accurate method) was used instead of confocal microscopy. Liao et al. used the preferred method of confocal microscopy and found that a pad conditioned at a higher downforce correlated to a smaller pad surface contact area and a higher silicon dioxide removal rate [3]. It is important to note that Liao et al. did not use confocal microscopy during the break-in process. Park et al. used a stylus-type surface profiler to analyze the pad surface for surface roughness throughout break-in [4]. Conditioner downforce was varied to determine the effect it may have had on the surface roughness, which was then successfully correlated to show that a higher surface roughness led to a higher blanket silicon dioxide RR. Surface roughness has been used as a common parameter to describe the polishing pad surface micro-texture in the past, however it describes the entire pad surface with one number and does not provide any insight as to how different aspects of the pad micro-texture are affected individually. Confocal microscopy was also used by Elmufdi et al. to determine the effect of conditioner type and downforce on pad-wafer contact area during break-in [5]. They discovered that increasing conditioner downforce led to an increase in pad-wafer contact area and further claimed that a higher contact area led to less CMP-related defects due to the higher contact imparting lower point stresses on the wafer. It should be noted that in their study, contact area was the only analyzed surface parameter and no topographic methods were used. In another study involving confocal microscopy Vasilev et al. investigated the effect of two different CVD-coated diamond discs on the evolution of pad surface roughness and pad surface height distribution during break-in [6]. They found that steady-state pad conditions could be reached within 20 min of break-in, for one disc, and within 50 min of pad break-in for the other [6]. It should be noted that their work did not report any relationships between pad break-in time and mean asperity (or summit) height, mean summit curvature, contact density, or percent pad-wafer contact area. Instead, their work relied on surface roughness parameters (i.e., S_a_, S_pk_ and S_vk_). Previous work by Sun et al. has demonstrated the importance of analyzing individual pad surface micro-texture parameters in more detail [7,8]. Metrics defining surface roughness are not the most effective descriptors of pad surface micro-texture in CMP simply because surface roughness represents the peaks as well as the valleys on the pad micro-texture yet CMP is a process that mostly involves contacting summits. As such, what should really matter is a measure of pad asperity height distribution, as well as contact area and density. This has been reported extensively to directly affect polish performance [2,3,4,5,6]. The results from the work of the above-mentioned researches presented a clear need to further develop a complete understanding of the pad-micro texture evolution throughout break-in due to different conditioner-downforce combinations, which was published in previous works by our group [9,10].

**Figure 1:**
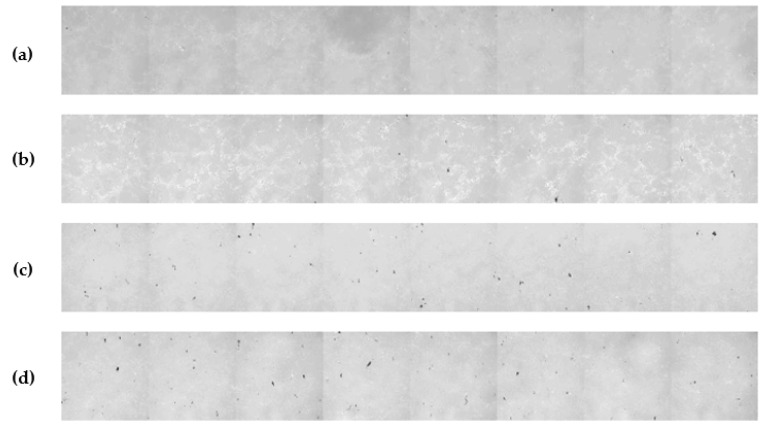
Contact images of (**a**) brand-new pad; followed by pads broken-in for 30 min with (**b**) EHWA disc at 2.7 kg_f_, (**c**) Abrasive Technology Incorporated (ABT) disc at 2.7 kg_f_ and (**d**) ABT disc at 0.9 kg_f_.

In previous works published by our group, McAllister et al. performed experiments where three different conditioner discs were used in conjunction with up to three different downforces to break-in separate brand-new pads from the same manufacturing batch [9,10]. Small samples were extracted from the center of the wafer track on each pad prior to break-in and after 5, 15, 30 and 60 min of break-in. This location is especially important since it contacts the wafer more frequently than other radial locations on the pad (i.e., due to longest dwell time). Confocal microscopy (CM) was then performed on land areas on the samples (i.e., between two grooves) to determine their micro-texture (in topographic mode) as well as their contact characteristics (in pressurized mode). The topographic and contact images acquired from CM were used to generate information on the surface height probability density function (PDF), mean summit height, mean summit curvature, as well as contact area and contact density. Details about the CM apparatus and the process of extracting the above metrics can be found elsewhere [7,8]. Results showed that in all cases, pad surface micro-texture parameters began to stabilize during the first 15 to 60 minutes of break-in. Two of the discs, one a conventional 3M disc and the other a CVD-coated EHWA (Irvine, CA, USA) disc, reacted to changes in downforce by producing very different pad micro-textures, and evolution paths, throughout the 60-minute break-in process. However, the conventional abrasive technology incorporated (ABT) disc produced very similar micro-texture results at 0.9, 2.7 and 4.5 kg_f_ of conditioning load. More important to the discussion to follow, McAllister et al. found that most of the contact area and contact density generated during the CMP process was due to fractured, poorly supported pore walls, which we refer to as pad fragments [9]. These pad fragments are described in great detail elsewhere [9], however they can be visually seen in the contact images of Figure 1 as black spots. In this work, we set out to correlate the CM results from our previous work with polishing performance immediately after breaking-in pads with different conditioner-downforce combinations.

## 2. Materials and Methods 

All tests were performed on an Araca APD-800 polisher and tribometer equipped with proprietary force transducers suitable for acquiring real-time shear and normal force at a frequency of 1000 Hz [11]. Average shear and normal force were found from the tens of thousands of instantaneous data points extracted from each wafer run in order to calculate the average coefficient of friction (COF) for each wafer run. It should be noted that the historical precision of COF on the APD-800 is within 5%. Three conditioner-downforce combinations were selected from our previous work to perform mini-marathon polishing tests using dozens of 200-mm blanket plasma enhanced tetraethylorthosilicate (PETEOS) wafers. Prior to any polishing, each of the 3 brand-new IC1000 K-grooved pads with Suba IV sub-pad (from the same batch manufactured by Dow Electronic Materials) having a diameter of 775 mm were broken-in for 30 min. This pad break-in time was used for each case in this study because the results from our previous work showed that the pad had reached a steady surface micro-texture within this time frame [9,10]. One brand-new pad was broken-in with the EHWA CVD-coated “1.3K” disc at 2.7 kg_f_, and two other brand-new pads were broken-in with the ABT S3410845N disc at 0.9 or 2.7 kg_f_. The ABT disc contained tens of thousands of “Type 3 1/2” diamonds randomly arranged in a ring-shaped (or donut-shaped) design on the outer edge of the discs working face. “Type 3 1/2” classification meant that the diamonds were sharper (with Type 5 being the sharpest) than they were blocky (with Type 1 being the blockiest). The diamonds had an average size of 173 μm which protruded above the substrate by 52 μm. In contrast, the EHWA disc contained 1300 micro-replicated tips which were uniformly machined across the full face of the disc. Each tip had a 45 × 45 μm^2^ square face and was 50 μm tall. These tips did not have a “Diamond Type” classification, however the square face indicated that they were more blocky than sharp. Additionally, the entire face of the EHWA disc was coated with a chemical vapor deposited (CVD) diamond carbon coating. During the break-ins, the platen and conditioning disc rotated counter-clockwise at 87 and 60 RPM, respectively. Ultra-pure water (UPW) was injected near the pad’s center at a flowrate of 250 mL/min and the conditioner swept across the pad 13 times per minutes. The same recipe was used for the in-situ conditioning during polishing, which was performed 100% of the time during polishing only. To mimic the actual process at Sandia National Laboratories, after pad break-in using the ABT disc, an ABT disc with a slightly different part number (S3410901N) was used during polishing and in-situ conditioning. During polishing, wafers were polished with a pressure of 4 PSI and a platen/carrier head RPM of 87/38. The slurry employed was Fujimi COMPOL-EX3 at 250 mL/min. The slurry contained colloidal silica-based nano-particles with an average size of 32.5 nm and a pH of 9.5, which was pre-diluted by a ratio of 7:4 (H_2_O:COMPOL-EX3). It should be noted that the different conditioner-downforce combinations were chosen only due to their enormous dissimilarities from one another. We hoped this would yield very different pad surface micro-textures and polishing results, and we never favored one combination over the other. 

## 3. Results and Discussion

The relative pad surface micro-texture results from our previous work after 30 min of break-in are shown in Table 1 for the three conditioner-downforce combinations used in this study.

**Figure 2:**
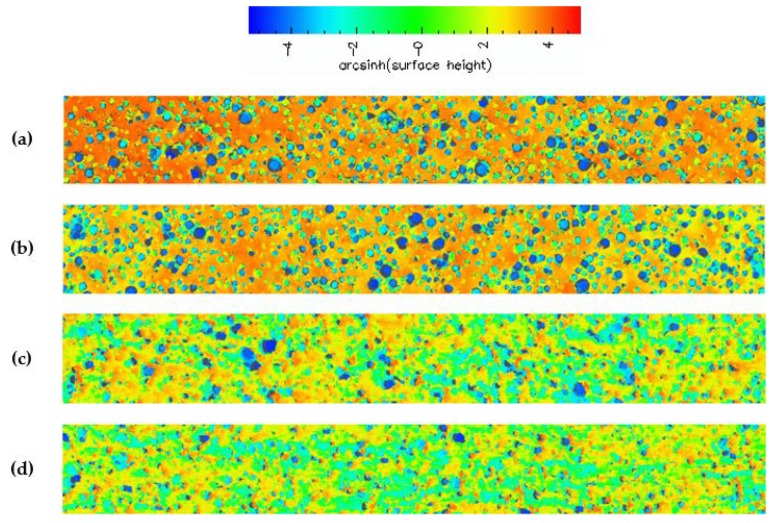
Surface topography of (**a**) brand-new pad; followed by pads broken-in for 30 min with (**b**) EHWA disc at 2.7 kg_f_, (**c**) ABT disc at 2.7 kg_f_ and (d) ABT disc at 0.9 kg_f_.

**Figure 3:**
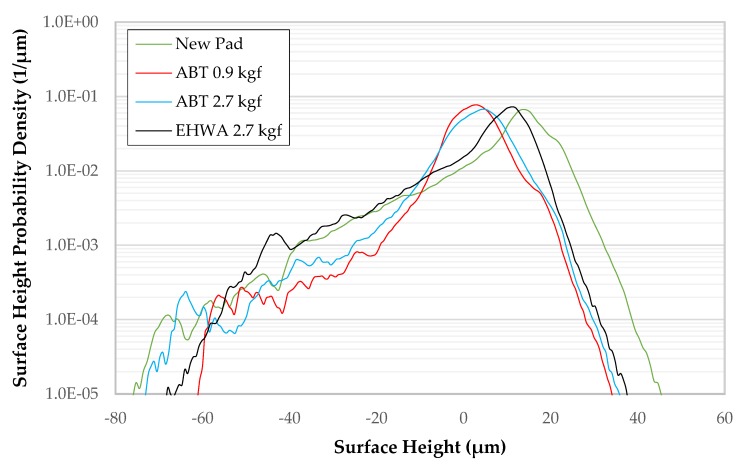
Surface height probability density functions (PDFs) corresponding to Figure 2.

Based on the summary of results in Table 1, the ABT disc produces flatter summits, more contact area and more contact density than the EHWA disc. These results are indicative of the ABT disc producing far more pad fragments than the EHWA disc [9,10]. To further support this claim, these pad fragments can be seen as black spots in the contact images from Figure 1. These fragments are much more prevalent on the pad broken-in with the ABT disc than the pad broken-in with the EHWA disc. In addition, these pad fragments are the reason for the decreased visibility of the pores (deep blue color) observed in the topography images of the pad broken-in by the ABT disc shown in Figure 2. This decreased visibility is caused by pore obscuration due to the collapse of the pore walls and the filling of the pores with pad debris as a result of the cutting of the pad by the conditioner disc. In order to gain further insight into pad micro-texture, we proceeded to quantify the images from Figure 2 by constructing summit height probability density functions (PDFs) as shown in Figure 3. The PDF of the pad surface is described in great detail in our previous work, however, in general the right-hand tail of the curve represents the highest parts of the surface (e.g., the asperities) while the left-hand tail represents the pores of the pad [9,10]. The slope of the right-hand tails is approximately the same for all three cases. Therefore, of more importance to this discussion is the left-hand tail of the PDF plot. The ABT disc generated nearly identical PDFs at both values of downforce, therefore the PDF of the ABT disc in general will be compared to the PDF of the EHWA disc. Comparing the slope of the left-hand tail of the PDFs, the slope for ABT becomes much steeper than for EHWA and the two left-hand tails completely separate. This is further indication of the pore obscuration that is present in the case of the ABT disc [10]. In comparison, the left-hand tail of the PDF generated by break-in with EHWA is closer to that of the left-hand tail of the PDF of the brand-new pad than it is to the PDF of the pad broken-in by ABT. This indicates that the EHWA disc is able to maintain the integrity of the pad pores during the break-in process, while the ABT disc clearly cannot. Therefore, the conclusion that ABT generates more pad fragments at both downforces than EHWA is valid. The reason behind this trend is likely due to the physical characteristics of the two types of discs. In the case of ABT, we believe that the increased sharpness, the larger average diamond size and the increased number of diamonds all conspire to increase the cutting efficiency of the disc. The ABT discs increased cutting efficiency of the pad surface contributes to the generation of more pad fragments as compared to the EHWA disc.

**Figure 4:**
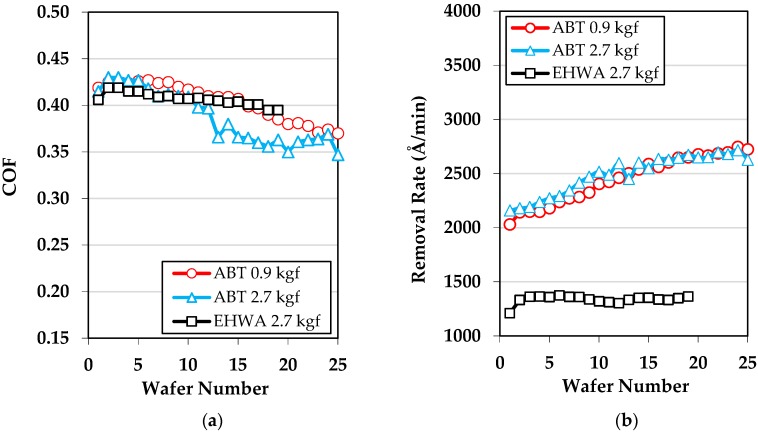
(**a**) Coefficient of friction (COF) and (**b**) removal rate (RR) results from the three mini-marathons.

After 30 min of pad break-in with each of the three conditioner-downforce combinations, we proceeded to polish up to 25 blanket SiO_2_ wafers. The coefficient of friction (COF) and removal rate (RR) results are shown in Figure 4. In a typical CMP process, RR increases with COF, yet the opposite trend is seen here for the ABT disc. For the EHWA disc, RR remains unaffected by a change in COF. There have been other instances where COF and RR have not been correlated, and, Borucki et al. [12] were able to explain the mechanism involved. They did so by isolating the individual contributions of abrasive nanoparticles that were caught on asperity tips and nanoparticles flowing through a nano-lubricated channel in-between the pad asperities and the wafer [12]. The mechanism is further explained using the results of this study, but before the results in Figure 4 can be explained, the two general types of sliding contact in CMP need to be explained. These are solid sliding contact and lubricated sliding contact. While both types of sliding contact are known to contribute to RR, only the solid sliding contact contributes to COF. This is because removal can occur as long as the slurry layer between the pad and wafer is thin enough to hold active slurry particles, which is the case in both types of sliding contact. In comparison, if there is no solid sliding contact, then minimal shear force will get generated which will have little contribution to friction. Therefore, when contacting summits are highly lubricated, COF and RR may become uncorrelated. This is in fact the case in this study. The pad fragments and the type of sliding contact can be used to explain the COF and RR data in Figure 4.

The ABT disc generates more pad fragments than EHWA as discussed above, leading to an overall higher RR for ABT. It is known that pad fragments are more easily lubricated because they are not fully supported and they generate a larger contact (i.e., higher surface area), therefore creating lower localized pressure and allowing more fluid to infiltrate the pad-wafer contact region [12]. This lubrication leads to a reduction in COF by causing a lower shear force. Therefore, when the ABT disc is switched from break-in to in-situ conditioning, more pad fragments are generated, causing COF to decrease throughout the mini-marathon. Simultaneously, the increase in pad fragments and contacting summits causes the RR to increase throughout the mini-marathon. From Figure 2 and Figure 3, the visibility into the pores for the pad broken-in with the ABT disc is clearly diminished compared to the pad broken in by the EHWA disc, however, this does not mean that the pad fragments are completely blocking the pores in which case RR would be diminished. For this reason, we believe that the pores are still able to assist in slurry transport and, as stated above, the lubricated pad fragments are causing the higher RR. In comparison, the EHWA disc generates similar COF results to the ABT disc, but the RR results are completely different. Here, the mechanism is similar except the EHWA disc generates less pad fragments than the ABT disc, therefore reducing RR. COF decreases slightly throughout the EHWA mini-marathon because of a slight pad fragment generation. This decrease is not as prominent as with ABT because EHWA creates much sharper asperities than the ABT disc. The sharper asperities and the negligible pad fragment generation during polishing keeps the RR steady during the mini-marathon with the EHWA disc. It should be noted within-wafer removal rate nonuniformity (WIWNU) did not change across the mini-marathon for all cases. In order to maintain the COF and RR both the amount of solid and lubricated sliding contact must both be maintained [12]. In this study the two types of sliding contact are changing by varying amounts, causing COF and RR to be uncorrelated.

## 4. Conclusions

It is important for a steady pad surface micro-texture to be reached before polishing with a brand-new pad can begin. The only means of achieving this, while minimizing the number of dummy wafers used, is through a proper break-in process with a diamond conditioner disc. The conditioner type and conditioner downforce directly impact the pad break-in micro-texture evolution process. In turn, the micro-texture generated during break-in plays an important role in determining the polishing performance. Using CM, micro-texture analysis in this study was implemented as a screening process for selecting the proper type of conditioning disc to be used. This was then used to optimize the polishing process during CMP which could ultimately result in a decrease in pad break-in time, and potentially an increase in pad life and a deeper understanding of the mechanism(s) behind the contact processes involved in a specific CMP process.

## Figures and Tables

**Table 1:** Micro-texture parameters relative comparison after 30 minutes of pad break-in.

Conditioning Disc	Down Force	Mean Summit Height	Mean Summit Curvature	Percentage Contact Area	Contact Density
ABT	X	X	X	4X	2.2X
ABT	3X	X	X	3X	1.6X
EHWA	3X	1.3X	2.4X	X	X
